# AI and the Future of Work: Assessing Occupational Social Status Perceptions Among University Students

**DOI:** 10.3390/bs16030362

**Published:** 2026-03-04

**Authors:** Jiawei Liu, Yifan Zhuang, Huaqi Yang, Siying Li, Chen Qu

**Affiliations:** 1 Key Laboratory of Brain, Cognition and Education Sciences, Ministry of Education, School of Psychology, Center for Studies of Psychological Application, and Guangdong Key Laboratory of Mental Health and Cognitive Science, South China Normal University, No. 55, West Zhongshan Avenue, Tianhe District, Guangzhou 510631, China; jiaweiliu19@scnu.edu.cn (J.L.);; 2 Faculty of Education, Northeast Normal University, 5268 Renmin Street, Changchun 130024, China

**Keywords:** occupational social status, artificial intelligent, threat perception, AI involvement

## Abstract

Artificial intelligence (AI) is profoundly reshaping labor market structures and occupational value evaluation systems. As a core group about to enter the workplace, university students’ perceptions of occupational social status are crucial for their career development and the alignment between education and the labor market. Study 1 explores how layoff risks and AI as a threat shape status evaluation. Study 2 investigates how AI’s role in jobs alters perceptions of status indicators and cognitive work type. The results show that students primarily attribute occupational social status to personal ability and organizational hierarchy rather than AI; A more positive attitude toward AI is associated with a greater propensity for pursuing routine cognitive occupations in the future; AI exerts an inverted U-shaped influence on occupational status indicators, with non-routine cognitive occupations experiencing an earlier decline in status but still maintaining higher ratings than routine cognitive occupations. These findings indicate that university students hold an overall positive yet contradictory attitude toward AI’s impact on occupational social status, which is inconsistent with actual employment trends. Therefore, researchers and policymakers should provide more comprehensive guidance to help students understand and adapt to AI-driven changes.

## 1. Introduction

The artificial intelligence (AI) technological revolution is profoundly reshaping social labor division and class structure, while reconstructing the traditional occupational value evaluation system (Tschang & Almirall, 2021). As a core group about to enter the workforce, college students’ perceptions of occupational social status in the AI era not only directly impact their personal career development and self-worth realization, but also have significant practical implications for future labor structure optimization, social labor division evolution, and the adaptive adjustment of education and training systems (Katsantonis & Katsantonis, 2024).

### 1.1. Theoretical Foundation and Measurement of Occupational Social Status

Guided by Weber’s theory of social stratification (Bowles, 2013), social status refers to the hierarchical level of prestige and respect an individual occupies within the social stratification system and in the perceptions of others (Kish-Gephart et al., 2023; Qu et al., 2017), measurable via objective quantitative assessments of socioeconomic status (e.g., educational attainment, work income) or subjective ratings with the MacArthur Ladder Scale (Adler et al., 2000; Kish-Gephart et al., 2023; Kraus et al., 2013). Compared with single indicators such as income or education, social status more comprehensively reflects college students’ subjective perceptions of occupations, and its formation and change directly affect future career decision-making.

In line with the talent cultivation in China’s higher education and the labor market demands, emerging fields such as the digital economy and high-end manufacturing increasingly require research and development-oriented and creative mental workers, while traditional labor-intensive positions are mostly replaced by technological upgrades or incorporated into the skill-based talent cultivation in vocational education (Chen et al., 2024).

Marked social status disparities exist across occupational categories, which classified and quantified into four types by job task nature: non-routine cognitive, routine cognitive, non-routine manual, and routine manual occupations (Dahlin, 2024). Non-routine cognitive occupations (e.g., financial analysts) depend on complex decision-making and professional expertise, and their task scarcity and irreplaceability make them hallmarks of upper-middle social status (Tschang & Almirall, 2021); routine cognitive occupations (e.g., counter staff) feature low skill barriers and high replaceability, aligning with lower-middle social status; non-routine manual roles (e.g., fitness coaching) demand adaptability but not advanced cognition; and routine manual jobs (e.g., food delivery) follow fixed procedures. A university degree used to be synonymous with a “respectable” cognitive occupations, but times have changed…

### 1.2. The Dual Impacts of AI on Employment and Occupational Cognition

In November 2022, OpenAI’s public release of ChatGPT, a generative AI (GenAI) tool with human-like conversational and practical capabilities, rapidly captured global attention. In 2023, ChatGPT was named among Nature’s top 10 scientific figures of the year. This marked the first time that a non-human entity had received such an honor, signifying that GenAI had officially established itself as a core technology driving social transformation. Within knowledge work contexts, GenAI’s enabling role primarily concentrates on three key areas: creative tasks in knowledge production, information processing tasks involving filtering and integration, and advisory tasks aimed at implementing and transforming solutions (Lee et al., 2025). Historically, these tasks were areas where college students excelled and were at the core of “respectable office positions”. Nowadays, these tasks are being rapidly replaced by GenAI, which raises graduates’ risk of post-graduation unemployment and underscores the need to dynamically adjust career expectations amid GenAI-driven labor market shifts.

However, AI wields a double-edged impact. Its dual effect of job replacement and technology empowerment is reshaping the current career ecology. From the displacement perspective, AI’s high efficiency at substituting routine, programmable and repetitive tasks drives employment structure “hollowing out” (Tschang & Almirall, 2021): it undertakes both standardized routine cognitive tasks but also assumes basic advanced cognitive work (e.g., financial data analysis, medical image screening), leaving practitioners vulnerable to job downsizing (Ivanov et al., 2020); these efficiency gains further trigger organizational adjustments, where enterprises curtail job demand and staffing to transition to a high-skill workforce paired with automation framework, directly squeezing middle and low-skilled positions’ survival space. Moreover, frequent employee-AI interactions trigger negative psychological responses such as loneliness and insomnia (Tang et al., 2023). In contrast, the complementary perspective highlights AI’s positive value around high-skill work empowerment and new job creation. AI imaging improves medical diagnostic efficiency by over 30% (Khalifa & Albadawy, 2024) and financial quantitative models enhance analysts’ data research capacity (Xing et al., 2025), reinforcing high-skill practitioners’ professional standing. AI has also spawned new occupations such as AI trainers and algorithm ethicists to fill tech governance talent gaps (Zhang et al., 2023). Moreover, AI streamlines organizational processes and reduces decision biases (Jago et al., 2024). In the education sector, it enables teachers to shift their focus from repetitive knowledge instruction to high value educational tasks such as cultivating students’ critical thinking (Gentile et al., 2023).

The in-depth integration of AI reshapes workplace and social cognition across status perception, power awareness, and technology acceptance. In terms of status perception, AI’s lower evaluation priority (vs. humans/computer) combined with job displacement threats and the “uncanny valley” effect creates a human–AI status gap (Ciechanowski et al., 2019). Furthermore, algorithmic management lowers perceived duty complexity and status ratings (Jago et al., 2024). This status misalignment exacerbates identity threats via status loss, as AI erodes professional authority and restructures work to cause career confusion (Mirbabaie et al., 2022). In terms of power awareness, AI shifts power cognition to technology tools (via humanized digital assistants), weakening constraints and raising manipulation risks (Fast & Schroeder, 2020). As for technology acceptance, social status exerts a notable impact on individuals’ acceptance of AI through education and income levels: higher-status groups possess stronger AI self-efficacy and operational proficiency, and high-income groups among them also have greater social persuasiveness in AI application (Hong, 2022). This group difference is specifically reflected in the service sector, where high-class groups show a clear preference for credit-based AI services (e.g., dental robots) over experience-based ones, with risk aversion acting as the key mediating factor behind this preference (Yao et al., 2022).

### 1.3. Research Gaps and Core Research Questions

Although research on AI and social status has accumulated certain achievements, most focus on the macro employment market, using large-sample questionnaire surveys or economic big data, and lack in-depth exploration of individual-level social status cognition, particularly ignoring college students’ perspective during the special stage of transition from campus to the workplace. We further summarize three core research gaps in existing studies: first, the lack of in-depth exploration of individual-level occupational social status cognition mechanisms; second, the shortage of targeted research on university students, a special group; third, the insufficient exploration of the differentiated interactive impacts of AI on the social status perception of different cognitive occupations. Notably, their projections of AI’s impact on occupational social status are of great significance for enhancing the rationality of their career decision-making and promoting the balanced development of the AI-era labor force structure.

Therefore, our research aims to systematically explore the impact of AI on university students’ perceptions of occupational social status through two interrelated experiments. Experiment 1 focuses on AI as a potential layoff risk variable, exploring its impact on students’ perceived threat to their personal occupational social status and its influence on their future career planning. Experiment 2 further manipulates the degree of AI involvement to examine its impact on students’ perceptions of related social status indicators and work types. We hypothesize that (H1) high AI-induced layoff rates would heighten university students’ perceptions of occupational status threat but not alter their core focus on personal ability in status evaluations; (H2) more positive AI attitudes would predict a stronger preference for routine cognitive occupations among students under high-skill conditions, owing to their risk-averse tendencies in career decision-making and underdeveloped proactive cognition of AI’s long-term substitution effects; and (H3) AI involvement would exert an inverted U-shaped effect on occupational status indicators, with non-routine cognitive occupations exhibiting earlier declines in status ratings than routine ones, driven by the shifting dominance of AI’s complementary and substitution effects across different involvement levels.

## 2. Experiment 1: Exploring University Students’ Perception of AI as a Threat to Social Status

### 2.1. Method

#### 2.1.1. Participants

A priori power analysis was conducted with G*Power 3.1.9 (Faul et al., 2007). With an effect size of 0.2, α = 0.05, and power = 0.95, including the interest of main effects and two-way interaction effects, a sample size of at least 30 participants were required, with an equal gender distribution. The actual sample size recruited for this study was 62 participants, which met the requirements for statistical analysis.

The 62 participants (32 females), aged 18–26 (M = 21.73, SD = 2.1), were recruited via posters, including students from South China Normal University and 17 from other higher education institutions in China, and received payment upon experiment completion. The South China Normal University ethics committee approved the study protocol.

#### 2.1.2. Experimental Design and Procedure

The purpose of Experiment 1 was to take AI as a potential threat factor of layoff risks, and investigate how university students’ confrontation with layoff risks in specific work scenario structures, incorporates their perceptions of the social status of their most likely occupation, perceived threats from factors including those in the current era context (such as AI), organizational structures and personal ability, and approaches to restructuring future career plans.

**Figure 1:**
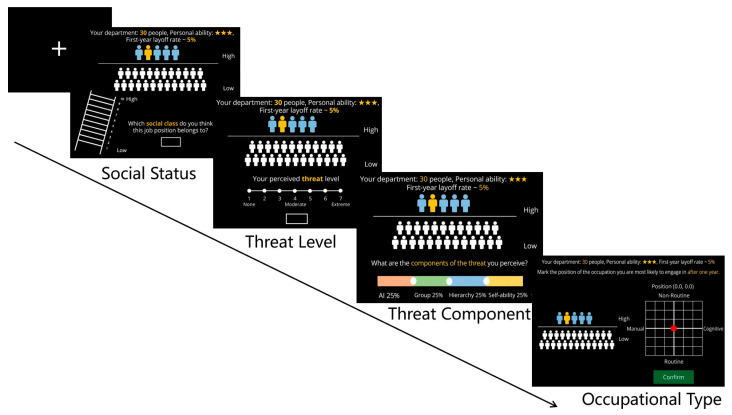
A flowchart of Experiment 1. Following the 1 s fixation point screen, all independent variables are displayed. The department size, the level of the participant’s personal ability, and the layoff rate in the first year are given at the top of the screen. The yellow avatar represents the participant themselves, the blue avatar represents the high-hierarchy group, and the white avatar represents the low-hierarchy group. The position of the yellow avatar on the screen changes accordingly based on the hierarchy level and the department size. As soon as the information is presented, the participant can make his or her decision. The decisions for the first two screens are completed by entering numbers into the input boxes. The decisions for the last two screens are completed by dragging the corresponding dots and then pressing the “Confirm” button.

When participants entered the laboratory, they first read and signed the Experimental Informed Consent Form, then explained the experimental procedure, task requirements, and conducted one practice trial with non-primary task materials. More precisely, the experimenter instructed participants to imagine the occupation they were most likely to pursue in the future and to immerse themselves in that scenario. Participants were then asked to drag the red dot to the corresponding position in the baseline coordinate screen, which had been presented prior to the initiation of the entire task. To facilitate comprehension, a printed document detailing the specific meanings of the four quadrants where x∈[−3,3] and y∈[−3,3], which described occupation types: non-routine cognitive (+, +), non-routine manual (−, +), routine manual (−, −) or routine cognitive (+, −), and listing related occupations was provided. The coordinate graph has the same right part of the career type interface as that in Figure 1.

The task adopted a 2 × 2 × 2 × 2 within-subject design, with the first-year layoff rate factor (5% verses 50%) as the anticipated probability of being dismissed within the next year, representing the macro-technological threat (the impact of AI on occupations is cyclical, and not all occupations will be affected simultaneously. The implementation of technology requires time, and one year is a quantifiable and reasonable duration (Benítez-Rueda & Parrado, 2024)); the number of employees in the department factor (department size: large number 80 versus small number 30) as the total number of individuals in the participant’s assigned work unit, serving as a structural indicator of organizational scale; the job hierarchy within the department factor (higher versus lower) as the participant’s relative status or position within the internal organizational structure, and personal ability factor (★★★ high-level versus ★ low-level) as the individual’s professional competence level assigned within the experimental scenario (Figure 1). Each trial consisted of four decision-making stages, each corresponding to a dependent variable. The first stage involved the question “Which social status do you think this job position belongs to?”, which measured job social status evaluation on a 1–10 point scale. The second stage was “To what extent do you perceive the threat?”, assessing perceived threat on a 1–7 point scale. The third stage was “Which threat components do you perceive?”, evaluating the proportion of threat components. Participants adjusted the proportional weights of four independent variables about ‘AI’, ‘Department size’, ‘Hierarchy’ and ‘personal ability’ by dragging, with the total summing to 100%. The fourth stage required participants to decide on the occupational type for the upcoming year within a four-quadrant coordinate system. The experimental trials were presented in a randomized order using PsychoPy-2022.2.5 (Peirce et al., 2019).

After participants completed Experiment 1, they were given a 5 min break before proceeding to Experiment 2. Finally, they completed a post-test involving self-reports regarding Experiment 1, which included two fill-in-the-blank questions: 1. “What specific occupation did you identify as the one you are most likely to pursue?”; 2. “What specific occupation did you identify as the one you are most likely to pursue in one year?”. They also filled out the Chinese version of the General Attitudes towards Artificial Intelligence Scale (CGAAIS) (Huang et al., 2025; Schepman & Rodway, 2023) simultaneously after finished Experiment 2.

The CGAAIS contained 15 items (1 attention check item, e.g., “I would appreciate it, if you could choose ‘strongly agree’”), including an 8-item positive attitude subscale (e.g., “Artificial intelligence can provide new economic development opportunities for the country”), and a 6-item negative attitude subscale (e.g., “I think artificial intelligence systems will make many mistakes”). The higher the score of each subscale, the more positive the attitude. Meanwhile, a high score on the negative subscale indicates a tolerant attitude towards the shortcomings of AI.

### 2.2. Statistical Analyses

We conducted statistical analyses by constructing a linear mixed-effects model (LMM) and created a base model which included the predictor variables Annual layoff rate, Department size, Job hierarchy and Personal ability, as well as their two-way interactions effects; the subject identity was fit as a random effect to control for repeated measures. Furthermore, we incorporated gender as an additional predictor into the full model to explore differences in perception of AI as a threat to social status. We compared the full model with the null model using a likelihood ratio test (LRT) to examine whether including gender as a predictor yielded a better data fit. Unless otherwise noted, base models provided a better fit to data than full models.

Here, we presented a base LMM formula with social status as the dependent variable:

$$social\;status\;\sim \;{\left(annual\;layoff+department+job\;hierarchy+personal\;ability\right)}^{2}+(1|subjects)$$

Other dependent variables included ‘threat level’ and ‘occupational type adjustment’, will also undergo comparative analyses of the base model and full model respectively. The ‘occupational type adjustment’ was analyzed from three perspectives: movement on the *x*-axis (i.e., manual-cognitive dimension), movement on the *y*-axis (i.e., nonroutine-routine dimension), and overall movement magnitude (i.e., z-diff, Euclidean distance), and they were subjected to z-score standardization prior to the LMM analyses to reduce inter-participant operational errors.

Regarding the threaten component variables of ‘AI’, ‘Department size’, ‘Hierarchy’ and ‘Personal ability’ involved in the threat factors, when the research focus is on the relative proportion of each part in the whole (e.g., the sum of proportions of AI and other roles equals 1), compositional data analysis (CODA) is a standard statistical method suitable for such data (Aitchison, 1982). When traditional statistical methods (e.g., linear regression) directly utilize raw proportional data, the “constant sum constraint” will lead to perfect multicollinearity among variables, which in turn induces biases in parameter estimation. By means of log-ratio transformations, such as isometric log ratio (*ilr*) (Egozcue et al., 2003), CODA converts compositional data into unconstrained new variables, fundamentally eliminating the influence of the constant sum constraint and ensuring that subsequent statistical analyses, such as multivariate analysis of variance (MANOVA) comply with model assumptions (Ferrer-Rosell & Coenders, 2017). The *ilr* transformation is one of the core techniques in CODA (Wester et al., 2025). Researchers can customize the composition of each factor according to specific theoretical hypotheses or research questions, a process known as Sequential Binary Partition (SBP) (Pawlowsky-Glahn et al., 2015). Specifically, the four-part threaten perception compositions of risk behavior of layoffs were transformed into three *ilr* coordinates using a SBP as follows: the first step is let the component vectors of the 4 threat factors be $x=(x_{1},x_{2},x_{3},x_{4})$, where

$x_{1}$ = AI_prop (proportion of AI threat),

$x_{2}$ = Depart_prop (proportion of Department size threat),

$x_{3}$ = Hierarchy_prop (proportion of Hierarchy threat),

$x_{4}$ = Skill_prop (proportion of Personal skill threat),

Then, the basic *ilr* coordinates used in this study were thus defined as

$${ilr}_{AI\_nAI}=\frac{\sqrt{3}}{2}\times \ln\left(\frac{x_{1}}{{\left(x_{2}x_{3}x_{4}\right)}^{1/3}}\right)$$

$${ilr}_{Ind\_Org}=\frac{\sqrt{2}}{3}\times \ln\left(\frac{x_{4}}{{\left(x_{2}x_{3}\right)}^{1/2}}\right)$$

$${ilr}_{InOrg}=\frac{1}{\sqrt{2}}\times \ln\left(\frac{x_{3}}{x_{2}}\right)$$

where ${ilr}_{AI\_nAI}$ represents the contrast of threat between AI and non-AI, analogously, ${ilr}_{Ind\_Org}$ indicates the threat trade-off between individual skills and organizational framework, ${ilr}_{InOrg}$ presents the threat of hierarchy level relative to the department size within the internal organizational structure. These unconstrained log-ratio transformations are compatible with conventional statistical analyses, while the derived results can be reverted to the original compositional space through inverse *ilr* transformation.

As explanatory variables, the MANOVA includes three *ilr* coordinates serve as dependent variables, jointly reflecting the relative proportion relationship of the four threat factors. After conducting the tests for multivariate normality and homogeneity of variance-covariance matrices (Box’s M test), we found that the null hypothesis was rejected. To ensure the validity of the MANOVA analysis, we used the robust Wilks’ Test, which combined the four threat factors into a multi-condition grouping variable of a one-way independent variable. Finally, through FDR multiple comparison correction, the overall effects of different experimental condition combinations on the three *ilr* dimensions were tested. All statistical analyses were conducted using R version 4.4.2, with packages including ‘lme4’, ‘lmerTest’, ‘performance’, ‘compositions’, ‘robustbase’ and ‘biotools’ employed for the analyses.

### 2.3. Results

#### 2.3.1. Occupational Social Status and Perception of Status Threat

We first analyzed a LMM analysis of the social status score with four factors: Annual layoff rate, Department size, Job hierarchy and Personal ability, as well as their interaction effects with the random effects of subjects. The results revealed significant main effects of job hierarchy (*b* = 1.31, SE = 0.19, t = 6.75, *p* < 0.001) and personal ability (*b* = 1.00, SE = 0.19, t = 5.15, *p* < 0.001), whereas no other significant effects were observed (Table S1). Specifically, individuals with higher job hierarchy or stronger personal ability were perceived to have higher social status than those in the corresponding low-level groups.

Next, we conducted another LMM analysis on the perception of layoff risk, incorporating the same main effects and interaction effects as mentioned above. The results showed significant main effect in layoff rate (*b* = 1.63, SE = 0.17, t = 9.57, *p* < 0.001), job hierarchy (*b* = −0.54, SE = 0.17, t = −3.17, *p* < 0.01) and personal ability (*b* = −0.67, SE = 0.17, t = −3.92, *p* < 0.001). These results indicate that perceived threat levels were significantly intensified under conditions of high layoff rates, low job hierarchy, and low personal ability compared to their respective alternative conditions. All other factors were not statistically significant (Table S2).

Further, we incorporated gender into the aforementioned base model and constructed a full model including gender and its interactions with all independent variables. The model comparison showed the full model fit the data better (χ^2^(5) = 18.99, *p* = 0.002). The results showed that consistent with the base model, the layoff rate, job hierarchy, and personal ability all exhibited significant main effects in the full model. Moreover, females perceived significantly higher threat than males (*b* = 0.904, SE = 0.241, t = 3.744, *p* < 0.001). The gender × layoff rate interaction was also significant (*b* = −0.441, SE = 0.170, t = −2.593, *p* = 0.01): females perceived significantly higher threat than males at low layoff rates (*p* < 0.001), no significant gender difference at high layoff rates (Table S3).

Subsequently, we performed CODA transformation on the proportional components of threat perceptions. After deriving the three *ilr* coordinates: ${ilr}_{AI\_nAI}$, ${ilr}_{Ind\_Org}$ and ${ilr}_{InOrg}$, we employed a one-way MANOVA to analyze the threat components. Here, the independent variable was a grouped variable (i.e., totaling 16 levels), and the dependent variables were the three *ilr* coordinates. The MANOVA results indicated a significant multivariate effect of the composite variable on the overall mean vector of the three *ilr* coordinates (Wilks’ λ = 0.7778, χ^2^(45) = 237.85, *p* < 0.001). To further explore the significant association, bonferroni correction was applied for post hoc multiple comparisons. Results showed that the proportional components of threat perception in “lower layoff rate + small-scale department + lower personal skills + higher hierarchy” context (5%.30.★.High, ${ilr}_{AI\_nAI}$ = −0.22, ${ilr}_{Ind\_Org}$ = 0.28, ${ilr}_{InOrg}$ = −0.13) differed significantly from those in the “high layoff rate + small-scale department + higher personal skills + lower hierarchy” context (50%.30.★★★.Low, ${ilr}_{AI\_nAI}$ = −0.35, ${ilr}_{Ind\_Org}$ = −0.2, ${ilr}_{InOrg}$ = −0.08). Specifically, in both contexts, the proportion of non-AI factors was higher than that of AI factors (${ilr}_{AI\_nAI}$). Regarding the proportional composition within internal organizational structures (${ilr}_{InOrg}$), the hierarchy factor was more significant than department size in both contexts. In the “5%.30.★.High” context, individual skills drove threat perception more strongly than organizational frameworks (${ilr}_{Ind\_Org}$)—a trend that reversed in the “50%.30.★★★.Low” context. Descriptive statistics revealed the following mean scores with relatively comparable standard deviations: AI (M = 20.65, SD = 10.17), Group size (M = 27.44, SD = 9.86), Hierarchy (M = 24.23, SD = 9.76), and Skill (M = 27.68, SD = 10.27).

After the experiment, we supplemented the collection of participants’ perceived other threats through a post-test questionnaire, and a total of 20 additional threat sources were gathered. Following further categorization, the collected responses were classified into three major threat factor categories: macro environment (52.2%, e.g., economic situation, legal environment), enterprise organization (26.1%, e.g., workplace interpersonal relationships, work pressure), and individual factors (21.7%, e.g., age, adaptability to work).

#### 2.3.2. Occupational Type Adjustment After One Year

**Figure 2:**
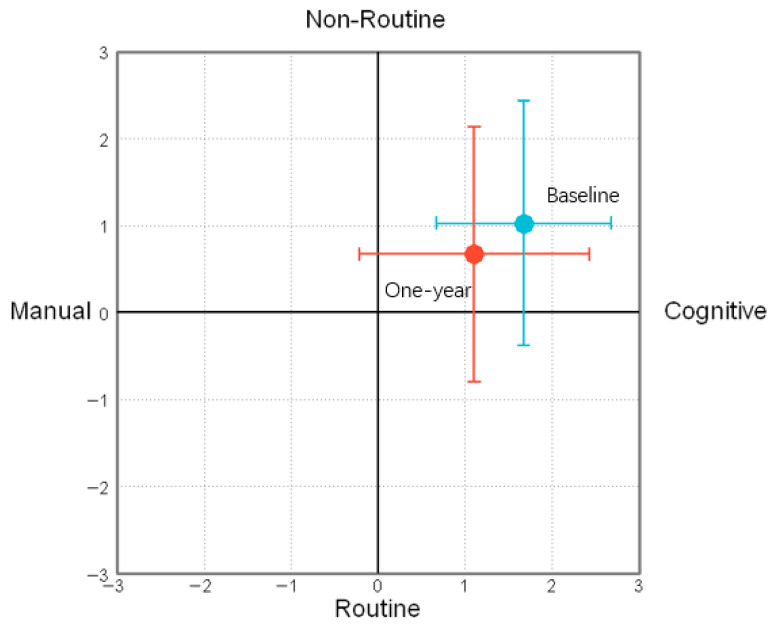
In Experiment 1, the average and standard deviation of the participants rated their personal occupational types in various situations (blue dots), and made adjustments to their career types one year later (red dots).

To estimate one-year changes in occupational type adjustment across different contexts, three separate LMMs were fitted to analyze the movement along the manual-cognitive dimension (*x*-axis), nonroutine-routine dimension (*y*-axis), and overall movement magnitude (z-diff). Figure 2 shows the average scores and standard deviations for participants’ self-assessed occupational types across various conditions, as well as the corresponding values adjusted one year later. For the *x*-axis (Table S4), significant main effects were observed for hierarchy factor (*b* = 0.46, SE = 0.13, t = 3.61, *p* < 0.001) and personal skills (*b* = 0.51, SE = 0.12, t = 4.04, *p* < 0.001), which individuals with higher hierarchy or greater skills showed a significantly stronger shift toward cognitive end than their lower-level counterparts. For the *y*-axis (Table S5), higher hierarchy (*b* = 0.35, SE = 0.14, t = 2.51, *p* = 0.012) and personal skills (*b* = 0.58, SE = 0.14, t = 4.12, *p* < 0.001) were significantly associated with a greater shift toward the nonroutine end. Regarding the overall movement range (z-diff, Table S6), significant main effects were found for layoff rate (*b* = 0.29, SE = 0.10, t = 2.90, *p* = 0.004), hierarchy (*b* = −0.33, SE = 0.10, t = −3.29, *p* = 0.001) and personal skills (*b* = −0.39, SE = 0.10, t = −3.89, *p* < 0.001). These findings demonstrate that future career adjustment was greater amid high layoffs, low hierarchy, and low skills compared to opposite conditions. No other factors showed significant effects in any of the LMMs.

Further, we expanded the base model for the z_diff by including gender and its two-way interactions with all predictors, which this full model demonstrated a significantly better fit (Table S7, χ^2^(5) = 16.41, *p* < 0.01). Beyond the already noted factors: layoff rate, job position, and personal skills remained significantly consistent, we detected a significant gender × layoff rate interaction (*b* = −0.316, SE = 0.099, t = −3.182, *p* = 0.002). Specifically, a high layoff rate led to a significantly larger adjustment magnitude among males (*b* = −0.41, SE = 0.07, t = −5.72, *p* < 0.001) but had no significant effect among females.

After Experiment 1, participants reported their most likely and expected occupation in a year. Over half (34) chose teaching, with programmer (only 4) as the second most popular. Remaining occupations (e.g., lawyer, designer) were highly concentrated in non-routine cognitive occupations.

To explore the relationships between university students’ attitudes toward AI, and adjustments to their future career planning across different contexts, we conducted a correlation analysis using the CGAAIS Scale. The total CGAAIS score averaged 55.98 (SD = 6.01). The mean score for the positive subscale was 32.22 (SD = 3.59), while the negative subscale had a mean of 23.77 (SD = 4.83). In the contexts of “50%.80.★★★.High” (r = −0.28, *p* = 0.03), “50%.30.★★★.High” (r = −0.29, *p* = 0.022), “5%.30.★★★.High” (r = −0.26, *p* = 0.04), and “5%.80.★.High” (r = −0.3, *p* = 0.018), the more negative university students’ attitudes toward AI, the more likely they were to choose routine occupations.

### 2.4. Discussion

The results indicated that occupational social status remained relatively stable across different contexts. Among the influencing factors, workplace hierarchy and personal ability emerged as core considerations in university students’ decision-making processes, indicating their tendency to attribute career-related threats to self-relevant dimensions. Even though the experimenter had explicitly informed participants of the independence between organizational hierarchy and personal ability prior to the experiment. An assertion corroborated by the CODA results showed that context-dependent threat attribution patterns were observed: in small-scale departments under low layoff risk, higher job hierarchy coupled with lower personal ability redirected participants’ threat perception toward personal ability; conversely, under high layoff risk, lower job hierarchy combined with higher personal ability shifted their threat perception to departmental hierarchy. This result indicated that university students recognize the essence of the social status lies in the judgment of its long-standing inherent value, the core capital for an individual to create value in a profession (Kish-Gephart et al., 2023). The job hierarchy is a product of the long-term evolution of social rules and organizational structures, with significant rigidity, and will not undergo fundamental changes due to short-term situations such as layoff risks (Andersson & Lindh, 2023).

Next, an analysis of changes in the score of social status threat and adjustments to career planning one year later revealed that, in addition to workplace hierarchy and personal ability, layoff rate also emerged as a critical factor influencing decision-making. The increase in layoffs rate will amplify the sense of job insecurity, intensify the concern about the possible decline of one’s social status, and thereby drive the adjustment of career planning. As can also be seen from the correlation results between the CGAAIS scale and the *Y*-axis, under the condition of high skills, the more positive participants are toward AI, the more they tend to prefer routine occupations. Although the scenarios designed in the experiment contained negative elements, which led university students to have a tendency for less efficient job positions and conservative choices in future career planning. Another interpretation is that low-skilled occupations provide bounded security via stable demand and low substitutability, functioning as a safe harbor during uncertainty (Abraham et al., 2018; Roys et al., 2019). In contrast, high-skilled sectors, despite their premium, are perceived as exposed to technological and cyclical risks (e.g., AI displacement, industry volatility) (Georgieff & Hyee, 2022). This risk differential may drive individuals toward conventional roles as a rational precautionary strategy (Huseynov, 2025).

The findings of Experiment 1 indicated that AI as a variable in the broader social context, which may not fully capture all the characteristics of the entire social environment. On the other hand, university students are still immersed in the school environment, and their perception of the threats posed by the real workplace environment remains insufficiently profound. Therefore, to explore how AI influences occupational social hierarchy, a more contextually appropriate individual-level perspective and explicit incorporation of AI as a variable is needed.

## 3. Experiment 2: AI Involvement’s Impact on Cognitive-Type Occupation

### 3.1. Method

Experiment 2 aimed to explore the impact of AI involvement on university students’ perception of the social status of cognitive occupations. To achieve this, 10 cognitive-type occupations and one occupation mentioned in Experiment 1 were selected as the research objects, and participants were asked to imagine scenarios where AI collaborated with these common occupations to complete work at different levels. Using a titrated AI involvement experimental paradigm, this study further supplements the precise influence of AI on social status cognition in Experiment 1, where AI served merely as a threat option and environmental context rather than a specific variable factor. The study systematically analyzed the differential impact paths of the degree of AI participation on social value judgments of occupations with different cognitive complexities through three observation dimensions: subjective perception of social status, objective socioeconomic indicators (educational level requirements and monthly income expectations), and evaluation of the types of core occupational tasks (creativity, advice, and information). The experimental hypothesis states that with the gradual deepening of AI’s participation in occupational work, university students’ perception of its influence on social status shifts from “cooperative empowerment” to “threat imposition”.

#### Experimental Design and Procedure

The subjects and devices were the same as in Experiment 1. The Experiment 2 adopted a 4 × 2 within-subject design, with the degree of AI involvement (0%, 25%, 50%, 75%), and the cognitive-type occupations factor include five non-routine cognitive jobs (financial analysts, lawyers, primary and secondary school teachers, AI technicians, translators), five routine-cognitive jobs (administrative clerks, bank tellers, salespeople, mail sorting clerk, tour guides), and the future careers participants might choose as referenced in Experiment 1 (the occupations chosen by participants will be categorized into relevant job types). These 10 occupations were chosen based on the previous research (Dahlin, 2024; Lee et al., 2025) and market salary level (Guangdong Provincial Department of Human Resources and Social Security, 2024), and for their accessibility and comprehensibility in daily life.

**Figure 3:**
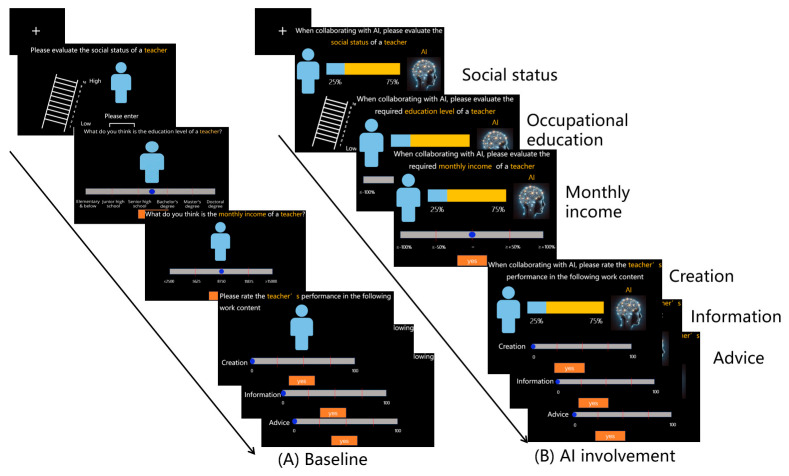
A flowchart of Experiment 2. (**A**) In the baseline condition, 11 occupations are presented at the top of the screen, and participants are required to evaluate six dimensions of the task. (**B**) In the AI involvement condition, the blue avatar represents the corresponding occupation, an AI agent is displayed on the right, and the bar indicates the degree of AI involvement in the job. Participants complete the task at a self-paced rate.

Firstly, participants were required to complete the baseline condition (Figure 3A), during which they were tasked with evaluating 11 occupations without the involvement of artificial intelligence, covering six specific dimensions: 1. An assessment of social status on a 1–10 scale; 2. Occupational educational requirements, categorized as primary school and below, junior high school, senior high school, undergraduate, master’s, and doctoral degrees; 3. Monthly income selection, with options ranging from CNY ≤2500 to CNY ≥15,000; 4. Creative rating task type, scored on a 0–100 scale; 5. Advice rating task type, scored on a 0–100 scale; 6. Information rating task type, scored on a 0–100 scale.

Upon completion of the baseline task, participants moved on to the task conducted under AI cooperation (Figure 3B). The degree of AI involvement in the occupation was randomly presented at three levels: 25%, 50%, and 75%. For the rating evaluation under the second dimension (Occupational educational requirements) and the third dimension (Monthly income selection), participants were required to compare the “AI involvement” condition with the baseline condition, using a progress bar ranging from −100% to 100%. The options for all other questions remained unchanged.

To facilitate comprehension, a printed document detailing the three categories and their sub-categories with examples for task type was provided, as well as two trials were conducted with non-primary task materials before the task, under both baseline and AI collaboration conditions. Finally, they completed a fill-in-the-blank question “What is your expected monthly salary after graduation?” regarding Experiment 2.

### 3.2. Statistical Analyses

Experiment 2 also adopted a base LMM which included the predictor variables AI involvement and cognitive type occupations factors with their two-way interactions effects, the subject identity was fit as a random effect to control for repeated measures. 

Here, we presented a base LMM formula with social status as the dependent variable:

$$social\;status\;\sim \;{\left(AI+cognitive\text{-}type\;occupation\right)}^{2}+(1|subjects)$$

Other dependent variables, including ‘Occupational educational requirements’ and ‘Monthly income selection’, ‘Creative rating task type’, ‘Advice rating task type’ and ‘Information rating task type’, will also undergo basic model analysis, respectively.

### 3.3. Results

#### 3.3.1. AI Influences Occupational Social Status

To examine the effects of occupational type (non-routine vs. routine) and AI intervention level (0%, 25%, 50%, 75%) on social status, we conducted separate LMM analyses on three dimensions: the overall score of occupational social status, the educational attainment requirements, and the monthly income of the occupation.

**Figure 4:**
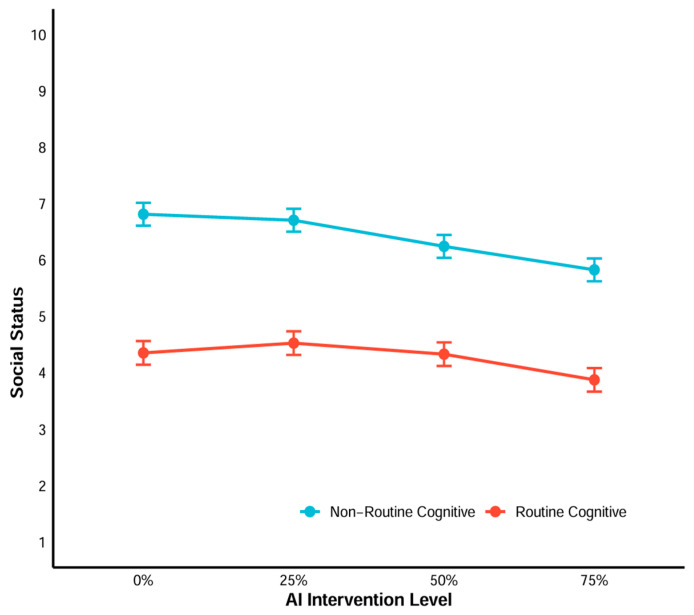
LMM results for the effects of occupational type and AI intervention level on social status.

First, the results of predicting subjective social status LMM are presented in Figure 4 and Table S8. For the main effects, routine cognitive occupations were associated with significantly lower social status compared to non-routine cognitive occupations (*b* = −2.46, SE = 0.10, t = −25.35, *p* < 0.001). Regarding AI intervention level, 50% and 75% intervention significantly reduced social status relative to 0% intervention, whereas 25% intervention showed no significant effect (*b* = −0.11, *p* = 0.25). All interaction terms between routine cognitive occupation and AI intervention level were statistically significant.

Post hoc comparisons highlighted distinct patterns between the two job types. For non-routine cognitive occupations (Table S9), social status decreased significantly with increasing AI intervention, with all higher intervention levels differing from lower ones. In contrast, routine cognitive occupations (Table S10) only showed no significant differences between 0% and 50% intervention (*p* = 0.99), nor between 25% and 50% intervention (*p* = 0.31).

**Figure 5:**
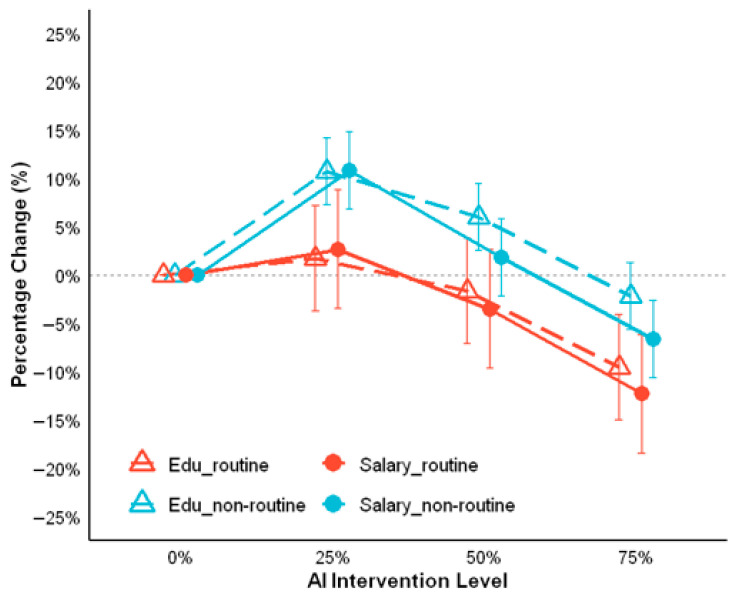
LMM results for the effects of occupational type and AI intervention level on Education attainment and Salary.

Second, the results of the LMM predicting educational requirements as the objective socioeconomic indicator of social status are presented in Figure 5 and Table S11. In terms of main effects, routine cognitive occupations exhibited notably lower educational requirements in comparison to non-routine cognitive occupations (*b* = −26.373, SE = 1.289, t = −20.454, *p* < 0.001). An inverted U-shape was observed for AI intervention between 0% and 50%, with performance significantly exceeding baseline levels, whereas 75% intervention level did not yield a significant effect (*b* = −1.709, *p* = 0.168). Similarly, there were significant interaction effects between routine cognitive occupation status and AI intervention at 25% and 50% levels, whereas 75% was not statistically significant. Next, the post hoc comparisons (Table S12) showed consistent non-significance for 0% vs. 75% AI intervention under non-routine type. Relative to other AI intervention levels, the 75% level yielded outcomes that were significantly different in routine cognitive occupation (0%: *p* = 0.002; 25%: *p* < 0.001; 50%: *p* = 0.018; Table S13). The pairwise comparisons between the other levels were not significant.

Last, the results of the LMM predicting monthly salary as the objective socioeconomic indicator of social status are presented in Figure 5 and Table S14. Routine cognitive occupations were associated with significantly lower salaries compared to non-routine cognitive occupations (*b* = −3649.88, SE = 216.73, t = −16.84, *p* < 0.001). Salary exhibited an inverted U-shaped pattern across AI intervention levels from 0% to 75%, peaking at an intermediate point. Notably, at the 50% intervention level, it did not differ significantly from the baseline (*b* = −739.48, *p* = 0.334). The interaction effect was only significant at 25% AI intervention level (*b* = −1011.794, *p* < 0.001). Then, the post hoc analyses (Table S15) showed 0% vs. 50% AI intervention was not significant for non-routine type (*p* = 1.00). Relative to other AI intervention levels, the 75% level yielded outcomes that were significantly different in routine cognitive occupation (0%: *p* < 0.001; 25%: *p* < 0.001; 50%: *p* = 0.02; Table S16). The pairwise comparisons between the other levels were not significant.

#### 3.3.2. AI Affects the Task Type of Occupations

We investigated how occupational type and AI intervention level influence the core work content characteristics of occupations—creativity, advice, and information-related tasks, which are also areas where AI excels by separate LMMs, in order to better explain changes in the occupations’ socioeconomic attributes.

**Figure 6:**
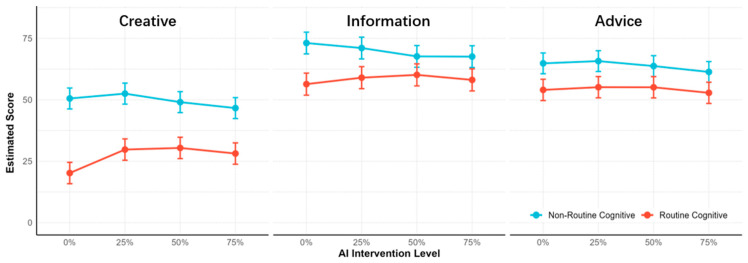
LMM results for the effects of occupational type and AI intervention level on Creative, Information and Advice task type.

First, the LMM for predicting creative task type in Figure 6 (left) and Table S17 showed routine cognitive occupations were associated with significantly lower creative requirements compared to non-routine cognitive occupations (*b* = −30.33, SE = 1.53, t = −19.86, *p* < 0.001). Regarding AI intervention level, only 75% intervention significantly reduced creative requirements relative to 0% intervention (*b* = −3.91, SE = 1.47, t = −2.67, *p* = 0.008), whereas 25% and 50% interventions relative to 0% showed no significant effects. Meanwhile, all interaction factors were statistically significant. Next, post hoc comparisons (Table S18) showed that for non-routine cognitive occupations, significant differences were limited to extreme levels: 75% < 25% (*p* < 0.001) and 75% < 0% (*p* = 0.046), other contrasts were non-significant. In contrast, routine cognitive occupations showed significantly lower creative requirements at 25%/50%/75% vs. 0% (*p* < 0.001 Table S19), with no differences between AI levels themselves.

Second, another LMM results for information task type in Figure 6 (middle) and Table S20 revealed that routine cognitive occupations had significantly lower requirements vs. non-routine (*b* = −16.72, SE = 1.38, t = −12.13, *p* < 0.001). AI 50%/75% reduced requirements vs. 0% (*p* < 0.001), 25% did not (*p* = 0.123). All interactions were significant. Then, post hoc comparisons (Table S21) highlighted contrasts, which only 50% and 75% AI intervention were associated with significantly lower requirements than 0% (*p* < 0.001) in non-routine occupation, no contrasts showed significant difference for routine occupations (Table S22).

Third, the LMM results for advice task type in Figure 6 (right) and Table S23 showed routine cognitive occupations had significantly lower requirements than non-routine ones (*b* = −10.80, SE = 1.74, t = −6.21, *p* < 0.001). Regarding AI intervention, only 75% reduced requirements vs. 0% (*b* = −3.50, SE = 1.67, t = −2.10, *p* = 0.036). All occupation and AI interaction factors were non-significant. Next, post hoc comparisons (Table S24) revealed a sole significant contrast in 75% < 25% (*p* = 0.049) for non-routine cognitive occupations. In contrast, no pairwise comparisons reached significance for routine cognitive occupations (Table S25).

#### 3.3.3. Attitudes Towards AI and Occupational Evaluations

For routine cognitive occupations, the positive subscale of the CGAAIS exhibited significant negative correlations with social status across all AI intervention levels (0%: r = −0.139, *p* = 0.014; 25%: r = −0.179, *p* = 0.001; 50%: r = −0.184, *p* = 0.001; 75%: r = −0.149, *p* = 0.019). In contrast, no significant correlations were detected between the CGAAIS and social status for non-routine cognitive occupations.

Next, across all AI intervention levels, significant correlations were found between the negative subscale of the CGAAIS (tolerant attitude) and monthly income for routine cognitive occupations: 0% (r = 0.158, *p* = 0.005), 25% (r = 0.368, *p* < 0.001), 50% (r = 0.240, *p* = 0.002), and 75% (r = 0.241, *p* = 0.002). In contrast, for non-routine cognitive occupations, the positive subscale of the CGAAIS was significantly correlated with monthly income solely under the condition of no AI intervention (0%: r = 0.114, *p* = 0.029).

Last, for both routine and non-routine cognitive occupations, the higher participants’ tolerance for AI, the lower they rated the performance of the information task type: routine (25%: r = −0.143, *p* = 0.011; 50%: r = −0.180, *p* = 0.001; 75%: r = −0.212, *p* = 0.001) and non-routine (25%: r = −0.170, *p* = 0.001; 50%: r = −0.202, *p* < 0.001; 75%: r = −0.152, *p* = 0.004).

In the post-test questionnaire, participants were asked about their expected monthly salary after graduation, and descriptive statistics of the collected data revealed a mean of CNY 10,695.2 ± 3805 (range: 3000–25,000).

### 3.4. Discussion

First, we found that non-routine cognitive occupations scored higher than routine ones in both subjective and socioeconomic measures of social status. As AI involvement increased, the status of non-routine occupations declined significantly, while routine occupations showed a slight, non-significant rise under low AI involvement before eventually dropping. But all indicators related to occupational social status showed a relatively small change (about 15%) when faced with the AI involvement (25–75%). This indicates AI impacts higher innovation occupations more strongly (Eloundou et al., 2024). For non-routine cognitive occupations, their core value lies in non-standardized innovation and decision-making tasks. Even if the degree of AI involvement increases moderately, it will trigger expectations of “the high-value aspects being replaced”, which will cause a decline in their social status evaluation at the early stage of AI intervention. For routine cognitive occupations, low-level AI involvement is often regarded as a productivity-enhancing tool, undertaking repetitive and cumbersome tasks, and helping practitioners optimize efficiency and reduce labor intensity. On the other hand, college students believed that the impact of AI on occupational social status is limited and has boundaries, and it does not cause a disruptive shock. This is similar to the result in Experiment 1, which is related to the relatively stable value of social status. Additionally, using a titrated AI involvement experimental paradigm, this study further supplements the precise influence of AI on social status cognition in Experiment 1, where AI served merely as a threat option and environmental context rather than a specific variable factor.

With AI intervention, routine cognitive occupations exhibited a consistent rating trend across the three tasks (creative, informational, and advisory), characterized by an initial increase, a late peak at approximately 75%, and a subsequent decline. For non-routine cognitive occupations, creative and advisory task ratings peaked earlier at around 25% then gradually decreased, while informational task ratings declined initially and stabilized thereafter. And the overall score for information task was the highest, followed by advice and creative. These findings indicate university students currently prioritize AI for information acquisition in occupational contexts, followed by content refinement and in-depth analysis, with such optimization supporting professional decision-making and planning (Qi et al., 2025). Notably, the five selected non-routine cognitive occupations lean toward traditional industries, where creative space and practice may have limited. By contrast, AI’s creative utility may gain greater recognition in humanities and arts (Zhou & Lee, 2024). Additionally, AI requires precise prompts and repeated adjustments when performing creative tasks, rendering its advantages less prominent compared to other task types (Oppenlaender et al., 2025).

Subsequently, correlation analysis between CGAAIS and social status indicators revealed significant occupational differences: university students with more positive AI attitudes showed lower social status evaluations but higher income expectations for routine cognitive occupations, while no such correlation existed for non-routine ones. This is due to the AI’s proficiency in performing the core tasks of routine occupations (e.g., data entry, basic copywriting), tasks that follow fixed rules, thereby reducing the perceived irrelevance (Dahlin, 2024). However, university students think AI-driven efficiency dividends and collaboration also contribute to wage premiums (Qi et al., 2025; Wang et al., 2022). In contrast, social status evaluation of non-routine cognitive occupations is anchored in creativity and complex decision-making capabilities that AI struggles to replace, hence showing no significant correlation with AI attitudes. Additionally, across all cognitive occupations, the higher individuals’ tolerance for AI, the lower their performance ratings of informational tasks. This means they have a better understanding of AI’s suboptimal performance in task processing, regard AI as a potential “professional tool,” and are more inclined to use it for informational tasks that test its accuracy and reliability (Qi et al., 2025).

## 4. General Discussion

This study systematically explored university students’ perceptions of occupational social hierarchies in an AI-enabled environment via two experiments. Specifically, the core finding of Experiment 1 was that students’ cognition of occupational social status remained stable even in scenarios involving potential AI threats and layoff risks, as their evaluations tended to be grounded in long-established social norms, organizational structures, job values, and individual competencies. However, a more positive attitude toward AI was correlated with a higher likelihood of students opting for less efficient occupations in their future career planning. Experiment 2 revealed that with deeper AI intervention, university students perceived non-routine cognitive jobs to suffer greater negative impacts than routine cognitive jobs, yet the relevant social status indicators of the former remained higher than those of the latter.

University students exhibit cognitive biases regarding the social status of cognitive occupations in the AI era. At the individual level, after long-term exposure to the campus evaluation system centered on competency, students tend to directly equate academic achievements with personal capabilities, forming a simplistic attribution logic that personal ability determines occupational value. They further apply this logic to occupational social status assessments, overlooking external variables like organizational resource allocation and technological disruptions. Second, university students perceive notable differences in social status attributes across cognitive occupations despite deep AI intervention and lean toward low-skill roles in career planning when facing layoff risks. More interestingly, when asked about expected post-graduation monthly salary, participants’ average expectation (CNY 10,695.2) was 1.7 times higher than domestic empirical survey data reported for undergraduates six months after graduation in 2024 (CNY 6199), showing a large discrepancy between university students’ expectations and actual outcomes (MyCOS Institute, 2025). However, a latest empirical study on the U.S. job market (Lichtinger & Hosseini Maasoum, 2025) found that the AI boom has triggered cross-industry job compression, with wholesale and retail (dominated by routine roles like clerks and sales assistants, which are highly AI-substitutable) being the hardest-hit sectors rather than internet or design industries. The research further found that AI-adopting firms saw a drastic decline in entry-level positions, while senior-level roles not only showed no such disparity with control group companies but also experienced stronger growth. The college students in this study tended to perceive an increase in job hierarchy levels during the initial stage due to the assistance of AI. They did not reflect the market trend of “reduction of entry-level positions and growth of senior-level positions”. This further confirmed that the career perception of college students has a distinct campus-centric feature, which is out of sync with the actual dynamics of the labor market. These findings suggest that universities or relevant authorities should provide more authentic labor market information and specialized career counseling. Such interventions are essential to help students cultivate a clearer awareness of the evolving occupational landscape in the AI era, enabling them to form more accurate professional value judgments and make better-informed career choices.

Historically, higher education was a key driver of intergenerational upward mobility (Hong, 2022; McGinn & Oh, 2017), but amid AI’s rapid advancement and college enrollment expansion, only graduates from top-tier institutions and specialized skill colleges retain strong job market competitiveness, while those from upper-middle-tier universities are most severely affected (Lichtinger & Hosseini Maasoum, 2025). China’s National Bureau of Statistics data (June 2024–May 2025) showed that the urban unemployment rate for laborers aged 16–24 (non-students) was around twice that of 25–29-year-olds and four times that of 30–59-year-olds. A domestic human resources service provider’s 2024 employee turnover report showed the overall rate was 15.3%, down 1.3 percentage points year-on-year. This reflects that enterprises’ talent demand has dropped significantly amid complex internal and external challenges, while employees’ preference for job stability has driven the continuous decline in turnover. All the above data point to a grim reality: as an augmented automation technology, AI can not only replace routine jobs (e.g., manufacturing and administrative positions), but it can also enhance production efficiency and create new roles through integration with technologies such as cloud computing and the Internet of Things. This has polarized the employment structure by a hollowing-out of middle-skill jobs while retaining high-skill and low-skill roles (Tschang & Almirall, 2021). The squeezed and replacement of formerly stable jobs have diminished workers’ economic security and precipitated a corresponding decline in their social status. The students’ cognitive biases, therefore, significantly underestimate the risks and challenges posed by this profound structural change when planning their careers: the perception that “low-skilled positions are safer” and the aspect of “personal ability determining one’s social status in the workplace”.

University students’ attitudes toward AI and their social status-related career decisions appear contradictory. Although they expect non-routine cognitive occupations to see an inverted U-shaped pattern in education requirements and income amid AI intervention, they simultaneously perceive the social status as declining steadily. Additionally, while students affirm AI’s life-improving value, they lower their ratings for social status indicators like income, job type, and future career plans. Admittedly, the formation of social status is a result of long-term accumulation. However, when technological revolutions drastically transform all industries, social status, a non-solidified attribute will lose its value at an accelerated pace. Recent academic perspectives mostly embrace AI’s augmentation value by highlighting its role in boosting individual productivity, yet such views often rely on narrow technological framing (e.g., exclusive focus on deep learning), anecdotal task-enhancement evidence, and abstract big data analysis (Tschang & Almirall, 2021). By contrast, in-depth interviews with front-line employees, coupled with experimental investigations, yield specific data on employee-related interests and scenarios, which is crucial for capturing AI’s impact on organizational capabilities and employment structures. For instance, in human resources fields, performing strategic tasks is a crucial manifestation of professionals’ expertise and social status; relying on AI may undermine their decision-making autonomy and threaten their professional identity. Without human involvement, AI will suffer from low social acceptability in complex non-routine tasks (Tinguely et al., 2023). Furthermore, another study comparing algorithmic management with typical humanized management also found that technological transformation predicts lower perceived hierarchical status compared to peers (Jago et al., 2024). These findings from workplace professionals align with the observations in this study regarding university students’ perception of AI-induced declines in occupational social status, thereby partially corroborating a broader association between technological disruption and status perception. Therefore, the perception of low status activates individuals’ “survival defense mode”, confining their cognitive focus to short-term interests and risk avoidance while neglecting long-term planning, which is further detrimental to university students’ employment and self-realization (Venkataramani et al., 2024). These findings suggest that when providing career guidance to university students, it may be beneficial not only to present the objective realities of the labor market but also to consider students’ potential social status anxiety. By fostering a perspective centered on AI-driven empowerment, educational institutions may assist students in navigating away from defensive cognitive orientations. Such an approach could help students view human–AI collaboration from the standpoint of long-term career development rather than perceiving it primarily as a threat.

Meanwhile, gender and layoff rate interacted significantly in predicting social status threat perception and one-year career adjustments: under high layoff rates, women reported stronger threats but fewer adjustments. In the context of the AI era, the differences in career perception between genders can also affect social status. A field study found that excessive male optimism in early job hunting raised their reservation wages and widened the gender pay gap (Cortés et al., 2023). However, as the process continued, men adjusted expectations per market feedback while women stayed stable, gradually narrowing the gap later. Another study analyzing mass layoff data in Denmark’s production sector found women faced higher unemployment risk and greater earnings loss than men in the first two years of unemployment, mainly due to differences in education level and child care (Ivandić & Lassen, 2023). The International Labour Organization global data shows that “3.7% of female employment globally is at risk of automation by generative AI, compared to only 1.4% for men”; this gap is wider in high-income countries, where “7.8% of women’s jobs are automation-prone—more than twice that of men (2.9%)” (Gmyrek et al., 2023). The report emphasizes that mismanaged tech transition could threaten decades of progress in women’s labor force participation due to job losses concentrated in female-dominated occupations.

AI frontier scientists are much more optimistic about AI’s future than the public (O’Donovan et al., 2025), and AI-proficient individuals have a competitive edge in the tech revolution, driving social status stratification as occupational value systems reconfigure in the AI era. University students need diversified career information and dynamic status metrics for optimal self-positioning, while research and policy sectors must prioritize fostering students’ learning/adaptability, building balanced career pathways, and correcting the “low status = high security” bias to align status perceptions with occupational risks and enhance career decision-making rationality (Acemoglu & Restrepo, 2018). Through an analysis of university students’ cognition regarding existing occupational social status in the AI era, this study provides targeted practical references for directing talent toward emerging sectors, mitigating structural employment conflicts, and supporting society in achieving a stable transition during technological shifts.

## 5. Limitations and Future Directions

The current study has several limitations warranting future research. First, its simulated workplace scenarios lack real pressures like market competition and other factors, and only set the environment context as AI, which may lead to response biases. Second, the sample is geographically concentrated (from Guangzhou, South China) and lacks discipline diversity, limiting result generalizability. Third, we began collecting participants in May 2025 and lasted for approximately one month. Due to the rapid iteration of AI technology, the time period for relevant experiments needs to be emphasized, and the results may various as time goes by. Future research could conduct long-term follow-ups of graduates, expand samples to diverse regions and majors, classify AI by application type to explore nuanced impacts, and combine subjective data with objective metrics like internship performance for more rigorous conclusions.

## Data Availability

The data of this study are available from the corresponding author on reasonable request.

## Supplementary Materials

The following supporting information can be downloaded at: https://www.mdpi.com/article/10.3390/bs16030362/s1.
