# Supplementary material for: AI and the Future of Work: Assessing Occupational Social Status Perceptions Among University Students
Source: Behav Sci (Basel). 2026 Mar 4;16(3):362. doi: 10.3390/bs16030362 (PMC13024095; doi:10.3390/bs16030362)
Supplement: Supplementary file 1 [file behavsci-16-00362-s001.zip › behavsci-4077180-supplementary.pdf]

Table S1. Linear mixed model with Layoff Rate, Department Size, Hierarchy, Personal Ability and their two-way interactions as the fixed effects and social status as the dependent variable in Experiment 1.

Fixed effect	Coefficient	SE	t-value	*p*
(Intercept)	4.218	0.193	21.819	<0.001
Layoff_50%	0.052	0.194	0.270	0.787
Size_Large	0.169	0.194	0.872	0.384
Hierarchy_High	1.310	0.194	6.746	<0.001
Skill★★★	1.000	0.194	5.148	<0.001
Layoff_50%:Size_Large	-0.121	0.194	-0.623	0.534
Layoff_50%:Hierarchy_High	-0.290	0.194	-1.495	0.135
Layoff_50%:Skill★★★	0.056	0.194	0.291	0.771
Size_Large:Hierarchy_High	0.379	0.194	1.951	0.051
Size_Large:Skill★★★	-0.226	0.194	-1.162	0.245
Hierarchy_High :Skill★★★	-0.024	0.194	-0.125	0.901

Note. Layoff_50% (reference level of layoff rate = 50%), Size_Large (reference level of department size = large), Hierarchy_High (reference level of hierarchy = high), and Skill★★★ (reference level of personal ability = high).

Table S2. LMM with Layoff Rate, Department Size, Hierarchy, Personal Ability and their two-way interactions as the fixed effects and perceived threat as the dependent variable in Experiment 1.

Fixed effect	Coefficient	SE	t-value	*p*
(Intercept)	4.098	0.163	25.205	<0.001
Layoff_50%	1.633	0.171	9.574	<0.001
Size_Large	-0.056	0.171	-0.331	0.741
Hierarchy_High	-0.540	0.171	-3.168	0.002
Skill★★★	-0.669	0.171	-3.924	<0.001
Layoff_50%:Size_Large	-0.012	0.171	-0.071	0.943
Layoff_50%:Hierarchy_High	0.101	0.171	0.591	0.555
Layoff_50%:Skill★★★	-0.077	0.171	-0.449	0.653
Size_Large:Hierarchy_High	-0.278	0.171	-1.631	0.103
Size_Large:Skill★★★	0.093	0.171	0.544	0.587
Hierarchy_High :Skill★★★	-0.069	0.171	-0.402	0.688

Note. Layoff_50% (reference level of layoff rate = 50%), Size_Large (reference level of department size = large), Hierarchy_High (reference level of hierarchy = high), and Skill★★★ (reference level of personal ability = high).

Table S3. The full LMM with Layoff Rate, Department Size, Hierarchy, Personal Ability, Sex and their two-way interactions as the fixed effects and perceived threat as the dependent variable in Experiment 1.

Fixed effect	Coefficient	SE	t-value	*p*

(Intercept)	3.631	0.202	17.948	<0.001
Layoff_50%	1.861	0.191	9.727	<0.001
Size_Large	-0.093	0.191	-0.486	0.627
Hierarchy_High	-0.385	0.191	-2.015	0.044
Skill★★★	-0.599	0.191	-3.130	0.002
sex(F)	0.904	0.241	3.744	<0.001
Layoff_50%:Size_Large	-0.012	0.170	-0.071	0.943
Layoff_50%:Hierarchy_High	0.101	0.170	0.593	0.553
Layoff_50%:Skill★★★	-0.077	0.170	-0.451	0.652
Layoff_50%:sex(F)	-0.441	0.170	-2.593	0.010
Size_Large:Hierarchy_High	-0.278	0.170	-1.637	0.102
Size_Large:Skill★★★	0.093	0.170	0.546	0.585
Size_Large:sex(F)	0.071	0.170	0.415	0.678
Hierarchy_High :Skill★★★	-0.069	0.170	-0.403	0.687
Hierarchy_High :sex(F)	-0.300	0.170	-1.766	0.078
Skill★★★:sex(F)	-0.137	0.170	-0.804	0.422

Note. Layoff_50% (reference level of layoff rate = 50%), Size_Large (reference level of department size = large), Hierarchy_High (reference level of hierarchy = high), Skill★★★ (reference level of personal ability = high), and sex(F) (reference level of sex = female).

Table S4. LMM with Layoff Rate, Department Size, Hierarchy, Personal Ability and their two-way interactions as the fixed effects and manual-cognitive dimension （x-axis） as the dependent variable in Experiment 1.

Fixed effect	Coefficient	SE	t-value	*p*
(Intercept)	-0.872	0.161	-5.410	<0.001
Layoff_50%	-0.248	0.128	-1.942	0.052
Size_Large	-0.017	0.128	-0.132	0.895
Hierarchy_High	0.460	0.128	3.609	<0.001
Skill★★★	0.515	0.128	4.036	<0.001
Layoff_50%:Size_Large	-0.111	0.128	-0.869	0.385
Layoff_50%:Hierarchy_High	0.083	0.128	0.654	0.513
Layoff_50%:Skill★★★	0.063	0.128	0.493	0.622
Size_Large:Hierarchy_High	-0.148	0.128	-1.157	0.247
Size_Large:Skill★★★	0.056	0.128	0.443	0.658
Hierarchy_High :Skill★★★	-0.168	0.128	-1.316	0.189

Note. Layoff_50% (reference level of layoff rate = 50%), Size_Large (reference level of department size = large), Hierarchy_High (reference level of hierarchy = high), and Skill★★★ (reference level of personal ability = high).

Table S5. LMM with Layoff Rate, Department Size, Hierarchy, Personal Ability and their two-way interactions as the fixed effects and nonroutine-routine dimension （y-axis） as the dependent variable in Experiment 1.

Fixed effect	Coefficient	SE	t-value	*p*
(Intercept)	-0.861	0.185	-4.666	<0.001
Layoff_50%	0.077	0.140	0.554	0.579
Size_Large	-0.014	0.140	-0.100	0.920
Hierarchy_High	0.350	0.140	2.505	0.012
Skill★★★	0.575	0.140	4.117	<0.001
Layoff_50%:Size_Large	-0.160	0.140	-1.142	0.254
Layoff_50%:Hierarchy_High	-0.262	0.140	-1.876	0.061
Layoff_50%:Skill★★★	0.127	0.140	0.906	0.365
Size_Large:Hierarchy_High	0.267	0.140	1.912	0.056
Size_Large:Skill★★★	-0.067	0.140	-0.482	0.630
Hierarchy_High :Skill★★★	0.136	0.140	0.976	0.329

Note. Layoff_50% (reference level of layoff rate = 50%), Size_Large (reference level of department size = large), Hierarchy_High (reference level of hierarchy = high), and Skill★★★ (reference level of personal ability = high).

Table S6. LMM with Layoff Rate, Department Size, Hierarchy, Personal Ability and their two-way interactions as the fixed effects and overall movement magnitude (z-diff) as the dependent variable in Experiment 1.

Fixed effect	Coefficient	SE	t-value	*p*
(Intercept)	0.207	0.110	1.879	0.061
Layoff_50%	0.290	0.100	2.904	0.004
Size_Large	-0.053	0.100	-0.527	0.599
Hierarchy_High	-0.328	0.100	-3.286	0.001
Skill★★★	-0.388	0.100	-3.886	<0.001
Layoff_50%:Size_Large	0.003	0.100	0.026	0.980
Layoff_50%:Hierarchy_High	-0.034	0.100	-0.343	0.732
Layoff_50%:Skill★★★	-0.058	0.100	-0.582	0.561
Size_Large:Hierarchy_High	0.078	0.100	0.786	0.432
Size_Large:Skill★★★	0.015	0.100	0.154	0.877
Hierarchy_High :Skill★★★	0.124	0.100	1.242	0.214

Note. Layoff_50% (reference level of layoff rate = 50%), Size_Large (reference level of department size = large), Hierarchy_High (reference level of hierarchy = high), and Skill★★★ (reference level of personal ability = high).

Table S7. The full LMM with Layoff Rate, Department Size, Hierarchy, Personal Ability, Sex and their two-way interactions as the fixed effects and overall movement magnitude as the dependent variable in Experiment 1.

Fixed effect	Coefficient	SE	t-value	*p*
(Intercept)	0.199	0.145	1.372	0.172
Layoff_50%	0.453	0.112	4.056	<0.001
Size_Large	-0.143	0.112	-1.278	0.201

Hierarchy_High	-0.261	0.112	-2.339	0.020
Skill★★★	-0.356	0.112	-3.183	0.002
sex(F)	0.016	0.184	0.085	0.932
Layoff_50%:Size_Large	0.003	0.099	0.026	0.979
Layoff_50%:Hierarchy_High	-0.034	0.099	-0.345	0.730
Layoff_50%:Skill★★★	-0.058	0.099	-0.585	0.559
Layoff_50%:sex(F)	-0.316	0.099	-3.182	0.002
Size_Large:Hierarchy_High	0.078	0.099	0.790	0.430
Size_Large:Skill★★★	0.015	0.099	0.155	0.877
Size_Large:sex(F)	0.175	0.099	1.760	0.079
Hierarchy_High :Skill★★★	0.124	0.099	1.250	0.212
Hierarchy_High :sex(F)	-0.130	0.099	-1.303	0.193
Skill★★★:sex(F)	-0.063	0.099	-0.633	0.527

Note. Layoff_50% (reference level of layoff rate = 50%), Size_Large (reference level of department size = large), Hierarchy_High (reference level of hierarchy = high), Skill★★★ (reference level of personal ability = high), and sex(F)(reference level of sex = female).

Table S8. LMM with AI intervention level, occupational type and their two-way interactions as the fixed effects and social status as the dependent variable in Experiment 2.

Fixed effect	Coefficient	SE	t-value	*p*
(Intercept)	6.814	0.103	66.444	<0.001
Routine_Cog	-2.459	0.097	-25.350	<0.001
AI_25%	-0.107	0.093	-1.148	0.251
AI_50%	-0.570	0.093	-6.121	<0.001
AI_75%	-0.986	0.093	-10.594	<0.001
Routine_Cog:AI_25%	0.280	0.137	2.041	0.041
Routine_Cog:AI_50%	0.547	0.137	3.992	<0.001
Routine_Cog:AI_75%	0.509	0.137	3.710	<0.001

Note. AI_25% (reference level of AI intervention level = 25%), AI_50% (reference level of AI intervention level = 50%), AI_75% (reference level of AI intervention level = 75%), and Routine_Cog (reference level of occupational type = routine-cognitive).

Table S9. Pairwise contrasts of social status between different AI intervention levels for non-routine cognitive occupations in Experiment 2.

Contrast	Estimate	SE	t-statistic	*p*
AI(25% - 0%)	-0.107	0.093	-1.148	1.000
AI(50% - 0%)	-0.570	0.093	-6.121	<0.001
AI(50% - 25%)	-0.463	0.093	-4.973	<0.001
AI(75% - 0%)	-0.986	0.093	-10.594	<0.001
AI(75% - 25%)	-0.879	0.093	-9.446	<0.001
AI(75% - 50%)	-0.416	0.093	-4.473	<0.001

Note. The column "Contrast" represents the difference in estimated marginal means between

the two specified AI intervention levels (e.g., "AI(25% - 0%)" indicates the value of the 25% level minus the 0% level).

Table S10. Pairwise contrasts of social status between different AI intervention levels for routine cognitive occupations in Experiment 2.

Contrast	Estimate	SE	t-statistic	*p*
AI(25% - 0%)	0.173	0.101	1.719	0.515
AI(50% - 0%)	-0.022	0.101	-0.223	1.000
AI(50% - 25%)	-0.196	0.101	-1.942	0.314
AI(75% - 0%)	-0.478	0.101	-4.743	<0.001
AI(75% - 25%)	-0.651	0.101	-6.461	<0.001
AI(75% - 50%)	-0.455	0.101	-4.520	<0.001

Note. The column "Contrast" represents the difference in estimated marginal means between the two specified AI intervention levels (e.g., "AI(25% - 0%)" indicates the value of the 25% level minus the 0% level).

Table S11. LMM with AI intervention level, occupational type and their two-way interactions as the fixed effects and education as the dependent variable in Experiment 2.

Fixed effect	Coefficient	SE	t-value	*p*
(Intercept)	76.874	1.349	57.006	<0.001
Routine_Cog	-26.373	1.289	-20.454	<0.001
AI_25%	8.231	1.238	6.651	<0.001
AI_50%	4.608	1.238	3.723	<0.001
AI_75%	-1.709	1.238	-1.381	0.168
Routine_Cog:AI_25%	-7.360	1.823	-4.037	<0.001
Routine_Cog:AI_50%	-5.457	1.823	-2.993	<0.001
Routine_Cog:AI_75%	-3.126	1.823	-1.715	0.086

Note. AI_25% (reference level of AI intervention level = 25%), AI_50% (reference level of AI intervention level = 50%), AI_75% (reference level of AI intervention level = 75%), and Routine_Cog (reference level of occupational type = routine-cognitive).

Table S12. Pairwise contrasts of education between different AI intervention levels for non-routine cognitive occupations in Experiment 2.

Contrast	Estimate	SE	t-statistic	*p*
AI(25% - 0%)	8.231	1.238	6.651	<0.001
AI(50% - 0%)	4.608	1.238	3.723	0.001
AI(50% - 25%)	-3.623	1.238	-2.928	0.021
AI(75% - 0%)	-1.709	1.238	-1.381	1.000
AI(75% - 25%)	-9.940	1.238	-8.031	<0.001
AI(75% - 50%)	-6.317	1.238	-5.103	<0.001

Note. The column "Contrast" represents the difference in estimated marginal means between

the two specified AI intervention levels (e.g., "AI(25% - 0%)" indicates the value of the 25% level minus the 0% level).

Table S13. Pairwise contrasts of education between different AI intervention levels for routine cognitive occupations in Experiment 2.

Contrast	Estimate	SE	t-statistic	*p*
AI(25% - 0%)	0.871	1.339	0.651	1.000
AI(50% - 0%)	-0.849	1.339	-0.634	1.000
AI(50% - 25%)	-1.720	1.339	-1.285	1.000
AI(75% - 0%)	-4.835	1.339	-3.612	0.002
AI(75% - 25%)	-5.706	1.339	-4.263	<0.001
AI(75% - 50%)	-3.986	1.339	-2.978	0.018

Note. The column "Contrast" represents the difference in estimated marginal means between the two specified AI intervention levels (e.g., "AI(25% - 0%)" indicates the value of the 25% level minus the 0% level).

Table S14. LMM with AI intervention level, occupational type and their two-way interactions as the fixed effects and salary as the dependent variable in Experiment 2.

Fixed effect	Coefficient	SE	t-value	*p*
(Intercept)	11157.389	225.665	49.442	<0.001
Routine_Cog	-3649.881	216.733	-16.840	<0.001
AI_25%	1209.618	208.040	5.814	<0.001
AI_50%	201.004	208.040	0.966	0.334
AI_75%	-739.484	208.040	-3.555	<0.001
Routine_Cog:AI_25%	-1011.794	306.453	-3.302	<0.001
Routine_Cog:AI_50%	-466.670	306.453	-1.523	0.128
Routine_Cog:AI_75%	-186.648	306.453	-0.609	0.543

Note. AI_25% (reference level of AI intervention level = 25%), AI_50% (reference level of AI intervention level = 50%), AI_75% (reference level of AI intervention level = 75%), and Routine_Cog (reference level of occupational type = routine-cognitive).

Table S15. Pairwise contrasts of salary between different AI intervention levels for non-routine cognitive occupations in Experiment 2.

Contrast	Estimate	SE	t-statistic	*P*
AI(25% - 0%)	1209.618	208.040	5.814	<0.001
AI(50% - 0%)	201.004	208.040	0.966	1.000
AI(50% - 25%)	-1008.614	208.040	-4.848	<0.001
AI(75% - 0%)	-739.484	208.040	-3.555	0.002
AI(75% - 25%)	-1949.102	208.040	-9.369	<0.001
AI(75% - 50%)	-940.488	208.040	-4.521	<0.001

Note. The column "Contrast" represents the difference in estimated marginal means between

the two specified AI intervention levels (e.g., "AI(25% - 0%)" indicates the value of the 25% level minus the 0% level).

Table S16. Pairwise contrasts of salary between different AI intervention levels for routine cognitive occupations in Experiment 2.

Contrast	Estimate	SE	t-statistic	*P*
AI(25% - 0%)	197.824	225.017	0.879	1.000
AI(50% - 0%)	-265.665	225.017	-1.181	1.000
AI(50% - 25%)	-463.489	225.017	-2.060	0.237
AI(75% - 0%)	-926.132	225.017	-4.116	<0.001
AI(75% - 25%)	-1123.956	225.017	-4.995	<0.001
AI(75% - 50%)	-660.466	225.017	-2.935	0.020

Note. The column "Contrast" represents the difference in estimated marginal means between the two specified AI intervention levels (e.g., "AI(25% - 0%)" indicates the value of the 25% level minus the 0% level).

Table S17. LMM with AI intervention level, occupational type and their two-way interactions as the fixed effects and creative task type as the dependent variable in Experiment 2.

Fixed effect	Coefficient	SE	t-value	*p*
(Intercept)	50.533	2.149	23.517	<0.001
Routine_Cog	-30.331	1.527	-19.863	<0.001
AI_25%	1.972	1.466	1.345	0.179
AI_50%	-1.490	1.466	-1.016	0.310
AI_75%	-3.912	1.466	-2.669	0.008
Routine_Cog:AI_25%	7.575	2.159	3.508	<0.001
Routine_Cog:AI_50%	11.699	2.159	5.419	<0.001
Routine_Cog:AI_75%	11.843	2.159	5.485	<0.001

Note. AI_25% (reference level of AI intervention level = 25%), AI_50% (reference level of AI intervention level = 50%), AI_75% (reference level of AI intervention level = 75%), and Routine_Cog (reference level of occupational type = routine-cognitive).

Table S18. Pairwise contrasts of creative task type between different AI intervention levels for non-routine cognitive occupations in Experiment 2.

Contrast	Estimate	SE	t-statistic	*P*
AI(0% - 25%)	-1.972	1.466	-1.345	1.000
AI(0% - 50%)	1.490	1.466	1.016	1.000
AI(0% - 75%)	3.912	1.466	2.669	0.046
AI(25% - 50%)	3.461	1.466	2.361	0.110
AI(25% - 75%)	5.883	1.466	4.014	<0.001
AI(50% - 75%)	2.422	1.466	1.652	0.592

Note. The column "Contrast" represents the difference in estimated marginal means between

the two specified AI intervention levels (e.g., "AI(25% - 0%)" indicates the value of the 25% level minus the 0% level).

Table S19. Pairwise contrasts of creative task type between different AI intervention levels for routine cognitive occupations in Experiment 2.

Contrast	Estimate	SE	t-statistic	*P*
AI(0% - 25%)	-9.546	1.585	-6.021	<0.001
AI(0% - 50%)	-10.210	1.585	-6.440	<0.001
AI(0% - 75%)	-7.931	1.585	-5.002	<0.001
AI(25% - 50%)	-0.663	1.585	-0.418	1.000
AI(25% - 75%)	1.615	1.585	1.019	1.000
AI(50% - 75%)	2.279	1.585	1.437	0.904

Note. The column "Contrast" represents the difference in estimated marginal means between the two specified AI intervention levels (e.g., "AI(0% - 25%)" indicates the value of the 0% level minus the 25% level).

Table S20. LMM with AI intervention level, occupational type and their two-way interactions as the fixed effects and information task type as the dependent variable in Experiment 2.

Fixed effect	Coefficient	SE	t-value	*p*
(Intercept)	73.089	2.224	32.863	<0.001
Routine_Cog	-16.722	1.378	-12.132	<0.001
AI_25%	-2.038	1.323	-1.541	0.123
AI_50%	-5.433	1.323	-4.107	<0.001
AI_75%	-5.524	1.323	-4.175	<0.001
Routine_Cog:AI_25%	4.668	1.949	2.396	0.017
Routine_Cog:AI_50%	9.200	1.949	4.721	<0.001
Routine_Cog:AI_75%	7.245	1.949	3.718	<0.001

Note. AI_25% (reference level of AI intervention level = 25%), AI_50% (reference level of AI intervention level = 50%), AI_75% (reference level of AI intervention level = 75%), and Routine_Cog (reference level of occupational type = routine-cognitive).

Table S21. Pairwise contrasts of information task type between different AI intervention levels for non-routine cognitive occupations in Experiment 2.

Contrast	Estimate	SE	t-statistic	*P*
AI(0% - 25%)	2.038	1.323	1.541	0.741
AI(0% - 50%)	5.433	1.323	4.107	<0.001
AI(0% - 75%)	5.524	1.323	4.175	<0.001
AI(25% - 50%)	3.395	1.323	2.566	0.062
AI(25% - 75%)	3.485	1.323	2.635	0.051
AI(50% - 75%)	0.091	1.323	0.069	1.000

Note. The column "Contrast" represents the difference in estimated marginal means between

the two specified AI intervention levels (e.g., "AI(0% - 25%)" indicates the value of the 0% level minus the 25% level).

Table S22. Pairwise contrasts of information task type between different AI intervention levels for routine cognitive occupations in Experiment 2.

Contrast	Estimate	SE	t-statistic	*P*
AI(0% - 25%)	-2.630	1.431	-1.838	0.397
AI(0% - 50%)	-3.767	1.431	-2.632	0.051
AI(0% - 75%)	-1.721	1.431	-1.203	1.000
AI(25% - 50%)	-1.137	1.431	-0.794	1.000
AI(25% - 75%)	0.909	1.431	0.635	1.000
AI(50% - 75%)	2.046	1.431	1.429	0.918

Note. The column "Contrast" represents the difference in estimated marginal means between the two specified AI intervention levels (e.g., "AI(0% - 25%)" indicates the value of the 0% level minus the 25% level).

Table S23. LMM with AI intervention level, occupational type and their two-way interactions as the fixed effects and advice task type as the dependent variable in Experiment 2.

Fixed effect	Coefficient	SE	t-value	*p*
(Intercept)	64.826	2.133	30.389	<0.001
Routine_Cog	-10.802	1.739	-6.212	<0.001
AI_25%	0.910	1.669	0.545	0.586
AI_50%	-1.105	1.669	-0.662	0.508
AI_75%	-3.499	1.669	-2.096	0.036
Routine_Cog:AI_25%	0.187	2.459	0.076	0.939
Routine_Cog:AI_50%	2.163	2.459	0.880	0.379
Routine_Cog:AI_75%	2.302	2.459	0.936	0.349

Note. AI_25% (reference level of AI intervention level = 25%), AI_50% (reference level of AI intervention level = 50%), AI_75% (reference level of AI intervention level = 75%), and Routine_Cog (reference level of occupational type = routine-cognitive).

Table S24. Pairwise contrasts of advice task type between different AI intervention levels for non-routine cognitive occupations in Experiment 2.

Contrast	Estimate	SE	t-statistic	*P*
AI(0% - 25%)	-0.9096	1.6693	-0.5449	1.0000
AI(0% - 50%)	1.1052	1.6693	0.6621	1.0000
AI(0% - 75%)	3.4986	1.6693	2.0959	0.2171
AI(25% - 50%)	2.0148	1.6693	1.2070	1.0000
AI(25% - 75%)	4.4082	1.6693	2.6407	0.0499
AI(50% - 75%)	2.3934	1.6693	1.4338	0.9105

Note. The column "Contrast" represents the difference in estimated marginal means between

the two specified AI intervention levels (e.g., "AI(0% - 25%%)" indicates the value of the 0% level minus the 25% level).

Table S25. Pairwise contrasts of advice task type between different AI intervention levels for routine cognitive occupations in Experiment 2.

Contrast	Estimate	SE	t-statistic	*P*
AI(0% - 25%)	-1.097	1.806	-0.607	1.000
AI(0% - 50%)	-1.058	1.806	-0.586	1.000
AI(0% - 75%)	1.196	1.806	0.662	1.000
AI(25% - 50%)	0.039	1.806	0.022	1.000
AI(25% - 75%)	2.293	1.806	1.270	1.000
AI(50% - 75%)	2.254	1.806	1.248	1.000

Note. The column "Contrast" represents the difference in estimated marginal means between the two specified AI intervention levels (e.g., "AI(0% - 25%)" indicates the value of the 0% level minus the 25% level).
